# Development and Characterization of Multiprincipal β‑Ti Alloys with Optimized Mechanical and Corrosion Properties for Biomedical Applications

**DOI:** 10.1021/acsomega.6c06327

**Published:** 2026-09-17

**Authors:** Rafael Formenton Macedo Santos, Virgilio Pereira Ricci, Pedro Akira Bazaglia Kuroda, Mariana Correa Rossi, Carlos Roberto Grandini, Carlos Alberto Della Rovere, Conrado Ramos Moreira Afonso

**Affiliations:** † Faculdade de Ciências, Laboratory of Anelasticity and Biomaterials, 28108 Universidade Estadual Paulista, Av. Eng. Luís Edmundo Carrijo Coube, 2085, 17033-360 Bauru, SP, Brazil; ‡ BioMet-CAM Laboratory, Department of Materials Engineering (DEMa), 67828 Universidade Federal de São Carlos, Rodovia Washington Luis Km 235, 13565-905 São Carlos, SP, Brazil; § Universidade Federal da Integração Latino-Americana, Foz do Iguaçu 85870-650, PR, Brazil; + USP - Lorena School of Engineering, University of São Paulo, 12602-810 Lorena, SP, Brazil

## Abstract

The development of novel β-titanium (β-Ti) alloys has become a promising strategy to overcome the intrinsic limitations of conventional biomaterials such as Ti–6Al-4 V and 316L stainless steel, particularly issues related to stress shielding and limited biocompatibility. In this study, equimassic β-Ti alloys (Ti–33Nb–33Zr, Ti–25Nb–25Zr–25Ta, and Ti–40Nb–40Zr) were designed, processed, and characterized with the objective of achieving an optimal balance between low elastic modulus and mechanical strength. The alloys were produced by arc melting and subsequently hot rolled, followed by comprehensive structural, thermal, and mechanical analyses. Microstructural characterization revealed a predominant β phase with minor α″ martensite, while both thermodynamic simulations and experimental results indicated β-transus temperatures below 500 °C. Among the investigated compositions, Ti–40Nb–40Zr exhibited the lowest elastic modulus (42 GPa), approaching that of human bone, combined with satisfactory mechanical strength and ductility (ultimate tensile strength: 791 MPa; elongation: 39%). In contrast, Ti–25Nb–25Zr–25Ta presented superior hardness (316 HV_0.5_) and enhanced thermal stability, highlighting its potential for load-bearing biomedical applications. Electrochemical assessments in simulated body fluid (SBF) demonstrated that all β-Ti alloys possess outstanding corrosion resistance, evidenced by low corrosion current densities, high polarization resistances, and the formation of stable passive films.

## Graphical abstract

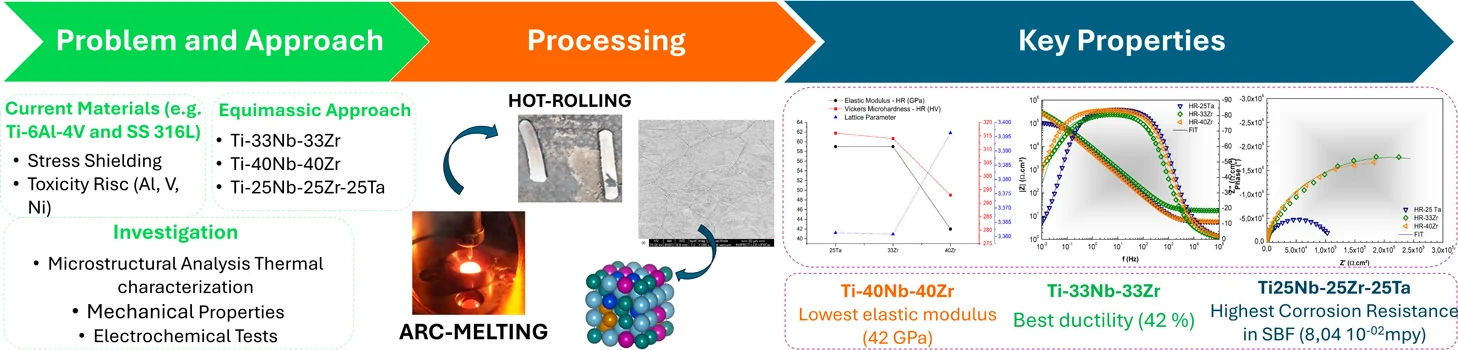

## Introduction

The development of new titanium alloys, such as beta titanium alloys (β-Ti), results from efforts to find a new material that does not exhibit the same issues as Ti–6Al-4 V (Ti-64) alloys and 316L stainless steel.^1–6^ Although Ti-64 and 316L alloys are widely used, they present two main problems: the mismatch between the elastic modulus of human bone and the implant (stress shielding), as well as biological incompatibility concerns, as the Nickel (Ni) in 316L is described as carcinogenic, and Aluminum (Al) in Ti-64 is associated with Alzheimer’s disease.^7–13^

Thus, the development of new materials with an elastic modulus closer to that of human bone (10–30 GPa), as well as composed solely of biocompatible elements, has been the motivation behind numerous studies.^14–21^ However, it is important to emphasize that fulfilling these two aspects alone is not sufficient for material to meet the requirements for use in implants. Among the other essential criteria, the material must exhibit excellent corrosion resistance in the human body, possess adequate mechanical strength, and, given the cyclic nature of movements, it must also demonstrate sufficient fatigue resistance.^1,3,21–24^

In recent years, an approach to increasing mechanical strength has involved equiatomic, non-toxic, multicomponent formulations with high entropy (BioHEA).^25–30^ However, although these alloys exhibit greater mechanical strength than traditional β-Ti alloys, such as Ti–40Nb, they also display a high elastic modulus, exceeding 140 GPa, as seen in the equiatomic Ti–Nb–Zr–Ta–Mo alloy.^27,28,30^ The high elastic modulus, compared to Ti–40Nb for example, is attributed to the high fractions of refractory elements such as Mo and Ta, which become predominant in these formulations due to their high atomic weight.^19^

In this regard, although it is common to find various alloys in literature with a reduced elastic modulus, for instance, lower than 90 GPa, it is much more challenging to identify alloys that combine biocompatibility, low elastic modulus, excellent corrosion and fatigue resistance, as well as good mechanical strength, when compared to the Ti64 alloy. The objective of this study was to develop β-Ti alloys with a low elastic modulus, good mechanical strength, and biocompatibility, with the potential to replace currently used alloys. To achieve this, an equimassic approach was proposed, which aims to significantly reduce the content of refractory elements, potentially resulting in alloys that combine a low elastic modulus with good mechanical strength, offering an improvement over the Ti64 alloy.

## Experimental Section

Initially, thermodynamic simulations, in equilibrium model, were carried out using ThermoCalc software, version 4.0 (2014), with the TCHEA5 database (a high entropy alloys database), to provide a “preliminary observation” of microstructural evolution as a function of temperature through the Property Diagram.

The equimassic alloys Ti–33Nb–33Zr (33Zr), Ti–25Nb–25Zr–25Ta (25Ta), as well as the Ti–40Nb–40Zr alloy (40Zr), were prepared through the addition of pure elements (>99.9%) in an arc melting furnace (Edmund Bühler D-72411 model arc melting furnace), under a protective argon atmosphere (as-cast condition, AC). Table 1 presents the chemical composition of these new multiprincipal BCC alloys in weight percentage, atomic percentage, and how the alloys will be referenced in this work.

**Table 1:** Chemical Composition of Multiprincipal BCC Alloys in Weight Percent (wt %), Atomic Percent (at. %), and Designation Used in this Work for the Alloys Studied

alloy (wt %)	alloy (at. %)	named in this work
Ti–25Nb–25Zr–25Ta	Ti_43.4_Nb_22.4_Zr_22.7_Ta_11.5_	25Ta
Ti–33Nb–33Zr	Ti_49.8_Nb_24.9_Zr_25.3_	33Zr
Ti–40Nb–40Zr	Ti_32.5_Nb_33.5_Zr_34.0_	40Zr

These alloys were subsequently thermomechanically processed via hot rolling, hot rolled condition, HR (conventional rolling mill, FENN brand, model 051). For the hot rolling process, the samples were heated in an electric resistance furnace at 900 °C for 15 min, without a protective atmosphere. The rolling process was then initiated. The thickness reduction per pass was 0.42 mm, and after every three passes, the samples were reheated in the furnace for 5 min to restore the temperature to 900 °C. Rolling was performed on all samples, referred to as the hot-rolled (HR) condition, until approximately 50% reduction in the cross-sectional area was achieved. Figure 1 presents photographs of a sample being prepared, a finished sample, and two samples after the hot rolling process.

**Figure 1:**
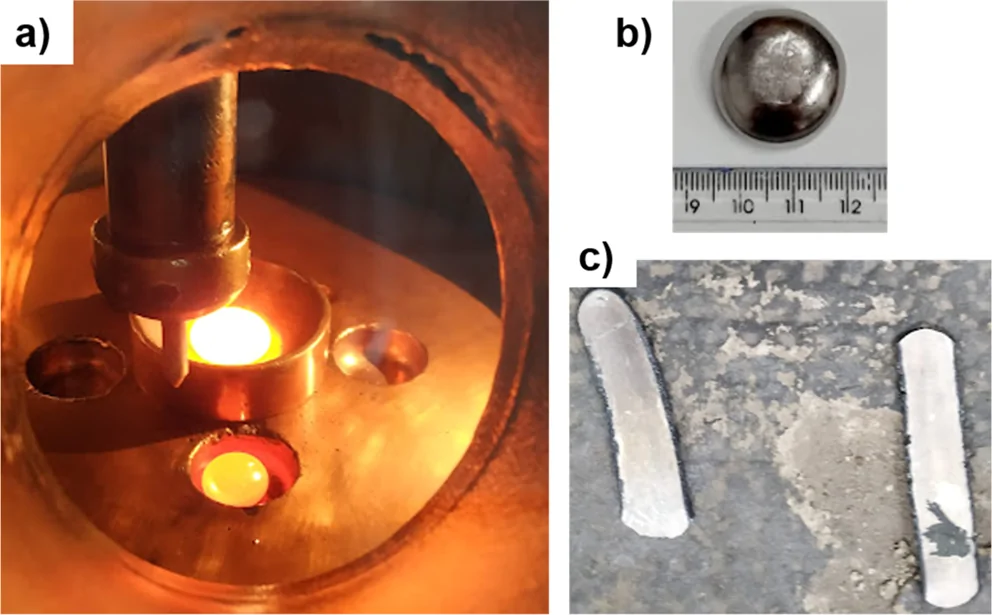
Images indicating (a) the alloy fabrication process in this work, (b) the finished as-cast sample (AC), and (c) the sample after the hot rolling process (HR).

As shown in Figure 1c, an oxide layer was observed on the rolled samples, which was subsequently removed through surface grinding.

Finally, they were characterized microstructurally, thermally, physically, mechanically, and electrochemical as outlined in the sections below.

- Microstructural characterization (HR condition): metallographic preparation (grinding up to 2000 mesh, followed by polishing with 1 μm alumina, and finally chemically etched with the traditional Kroll reagent); optical microscopy (OM - Olympus, model BX41M-LED); scanning electron microscopy (FEG-SEM - Philips XL30) and EDS (Oxford Link Tentafet X-ray detector); transmission electron microscopy (Tecnai G2 F20); oxygen content determination (LECO RO 400) and, X-ray diffraction (XRD) analysis was conducted using Cu–Kα radiation (λ = 1.5418 Å) over a scanning range of 20° to 90° with a step size of 2°/min (Rigaku, Geigerflex ME210GF2 model). Based on the XRD diffraction peaks, the lattice parameters of the beta phase (BCC) were determined using Bragg’s law and the equation for calculating interplanar distances.^16,31,32^

- Physical and mechanical characterization (HR condition): elastic modulus determination via impulse excitation (ATCP Sonelastic) according to ASTM E1876:2001.^33^ Vickers microhardness (Shimadzu, model HMV-G 20ST) was determined by averaging 15 measurements per sample, using a load of 0.5 kgf applied for 15 s, in accordance with ASTM E384-17.^34^ The density was determined using Sonelastic software, which calculates this property based on the mass-to-volume ratio. Tensile tests were also performed according to ASTM E8/E8M using miniaturized specimens, with an Instron 5500R universal testing machine.^35^ Since the elastic modulus is a key property in this study, optical extensometry was employed to obtain a more precise measurement of the elastic modulus. Six samples, in the rolled condition, were tested for each of the 33Zr, 40Zr, and 25Ta alloys. It is important to highlight that the fracture analysis was conducted using SEM in Secondary Electron (SE) mode to investigate the cause of the fracture.
- Thermal characterization (HR condition): Thermodynamic simulations were performed using Thermo-Calc, employing the TCHEA5 database, which is designed for high-entropy alloys. These simulations aimed to provide a preliminary assessment of microstructural evolution as a function of temperature through the Property Diagram, where the *x*-axis represents temperature and the *y*-axis represents the volume fraction of all phases (m^3^). The simulations were conducted under equilibrium conditions. For DSC tests, initially, the samples underwent heat treatment at 900 °C for 6 h, followed by slow cooling (in the furnace), enabling the detection of the β (BCC) → α (HCP) transformation (β-transus) during the test. Subsequently, 10^–2^ g of each sample was heated at a rate of 10 K/min, with data collected from 25 to 700 °C, using the Netzsch DSC 404 equipment.
- Electrochemical characterization (HR and AC condition): the corrosion resistance evaluation was performed using a Gamry Reference 3000 system integrated into a conventional three-electrode electrochemical setup, comprising a platinum counter electrode, a silver/silver chloride reference electrode (Ag/AgCl in saturated KCl), and cylindrical samples as working electrodes. The samples, machined by electric discharge machining (EDM) with an exposed surface area of 0.126 cm^2^, were electrically connected using a copper wire prior to embedding in resin. To accurately simulate physiological conditions, all electrochemical tests were conducted in the absence of light, using Simulated Body Fluid (SBF, pH 7.4, 36.5 °C) as the electrolyte.^36^ The electrochemical characterization followed five sequential steps: (1) initial cathodic polarization at −1000 mV_Ag/AgCl_ for 120 s to minimize the influence of the native oxide film on the working electrode surface; (2) monitoring of the open-circuit potential (OCP) over 3600 s (1 h) immersion in SBF, formulated to replicate the ionic composition of human blood plasma (Na^+^ 142.0, K^+^ 5.0, Mg^2+^ 1.5, Ca^2+^ 2.5, Cl^–^ 147.8, HCO_3_^–^ 4.2, HPO_4_^2–^ 1.0, SO_4_^2–^ 0.5 mM) [1]; (3) electrochemical impedance spectroscopy (EIS) performed immediately after OCP measurement within a frequency range of 100 kHz to 0.01 Hz using a sinusoidal excitation amplitude of 10 mV at OCP; (4) reassessment of the OCP after 300 s immersion in SBF; and (5) potentiodynamic polarization was performed from −500 mV with respect to the OCP up to +3000 mV_Ag/AgCl_, at a scan rate of 1 mV/s, to further evaluate the corrosion resistance. Although the upper potential limit used exceeds typical in vivo OCP values which are significantly lower than +3000 mV_Ag/AgCl_ a higher and more conservative limit was intentionally chosen to induce and analyze the onset of localized corrosion phenomena in the tested samples.^37^ For each condition, at least three independent replicates were conducted to ensure reproducibility and statistical reliability of the results; therefore, only representative curves for each condition are presented in this study.

Although this article presents the microstructural, mechanical, and physical characterizations of the HR condition, comparable data for the AC condition are available in literature.^16,38^ Regarding the electrochemical and corrosion characterizations, this study provides the corresponding results for both conditions, AC and HR.

## Results and Discussions

### Thermal Characterization

Figure 2 provides an in-depth analysis of the phase transformations and thermal behavior of the 25Ta, 33Zr, and 40Zr alloys, combining thermodynamic simulations (using ThermoCalc, database TCHEA5) and experimental DSC data. These results highlight critical temperature ranges, phase stability, and decomposition processes, emphasizing the influence of alloy composition on phase evolution and thermal properties.

**Figure 2:**
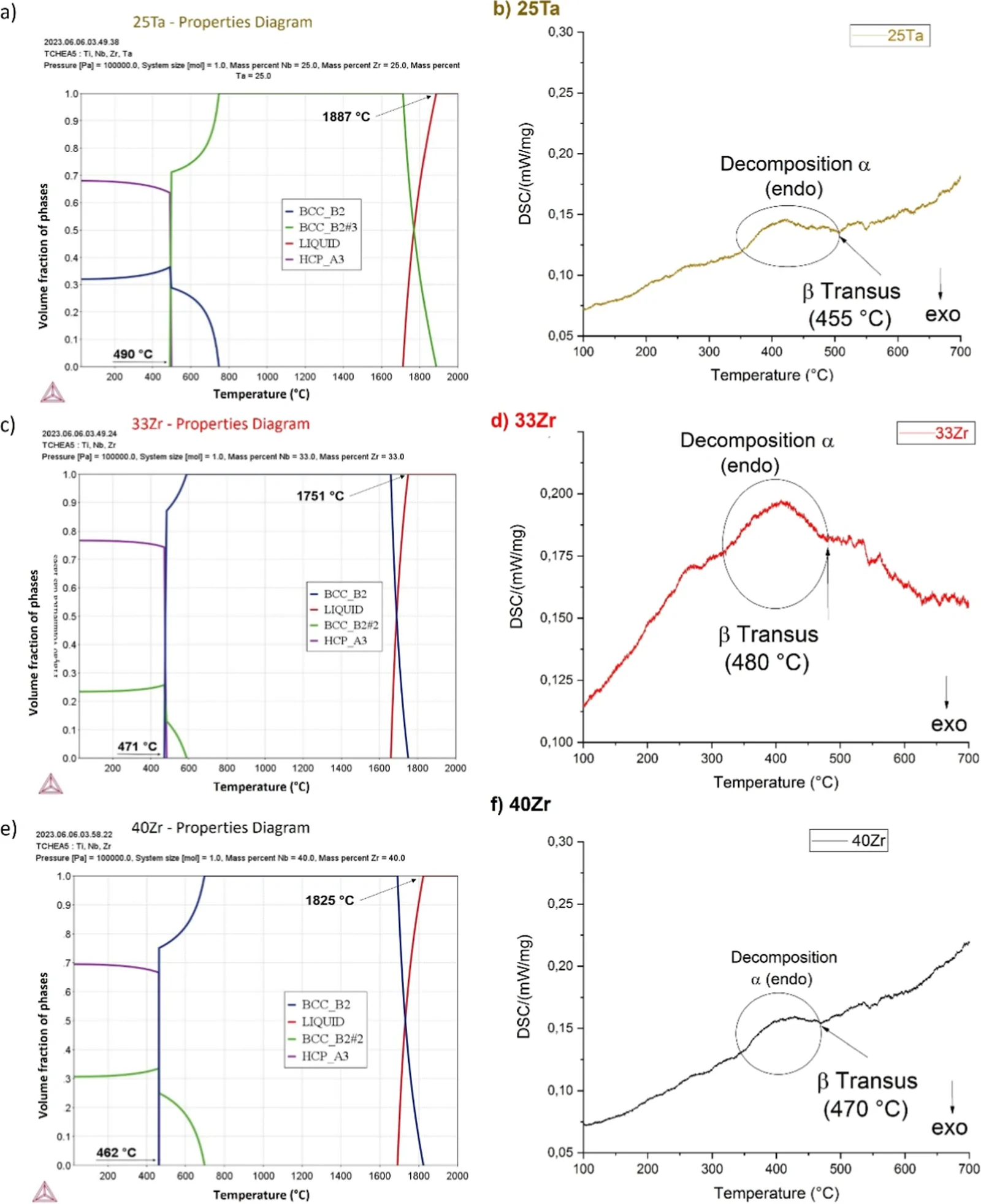
Property Diagrams Obtained Using ThermoCalc Software (Database TCHEA5) Under Equilibrium Condition as well DSC thermogram for the Alloys: (a,b) 25Ta; (c,d) 33Zr, and (e,f) Ti40Zr.

The ThermoCalc property diagrams predict a consistent sequence of phase evolution for all three alloys, with similar phase formation trends. However, variations in the β-transus temperature and phase coexistence regions are evident, directly linked to the differing chemical compositions of the alloys. For instance, the β-transus temperatures predicted by ThermoCalc are 490 °C for 25Ta, 471 °C for 33Zr, and 462 °C for 40Zr. These simulated values align closely with experimental results obtained via DSC, where the β-transus temperatures are 470 °C, 480 °C, and 455 °C, respectively. This agreement underscores the robustness of thermodynamic simulations in predicting phase stability, although the minor discrepancies between experimental and simulated values arise from the non-equilibrium heating conditions during the DSC tests, conducted at a rate of 10 °C/min.

The DSC thermograms further reveal key features of the alloys’ thermal behavior, particularly the presence of endothermic peaks associated with phase decomposition events. These peaks, observed between 450 °C and 480 °C, correspond to the decomposition of the α phase (α → β). This transformation is a critical indicator of the thermal stability of the β phase in these alloys, reinforcing their potential for applications requiring stability at elevated temperatures. Notably, the endothermic peaks’ proximity to the β-transus temperatures identified experimentally validates the DSC results and aligns with the thermodynamic predictions.

Table 2 presents the liquidus and β-transus temperatures obtained via Thermo-Calc, as well as the β-transus temperature determined by DSC.

**Table 2:** – Liquidus Temperature and β-Transus Temperature Obtained via ThermoCalc and β-Transus Obtained via DSC

alloy	liquidus temperature (°C) thermoCalc	β-Transus (°C) thermoCalc	β-transus (°C) DSC
25Ta	1887	490	470
33Zr	1751	471	480
40Zr	1885	462	455

A detailed examination of the 33Zr alloy property diagram reveals intricate phase transitions across various temperature ranges. Below 471 °C, a narrow coexistence of β, β′, and α phases is observed, emphasizing the thermodynamic complexity near the β-transus region. Between 471 °C and approximately 595 °C, the material stabilizes into β and β′ phases, transitioning to a single β phase above 595 °C. The coexistence of β and liquid phases is predicted between 1670 °C and the melting point at 1751 °C. These observations underline the critical role of temperature in determining the stability and coexistence of phases in the 33Zr alloy.

The 25Ta and 40Zr alloys exhibit similar trends in phase evolution but with subtle differences attributable to their compositions. The 25Ta alloy, with a β-transus temperature of 490 °C and a melting point of 1887 °C, displays a broader temperature range for β phase stability, indicating its structural robustness. Conversely, the 40Zr alloy, with a β-transus at 462 °C and a melting point of 1885 °C, shows a slightly narrower β phase stability range but compensates with a lower elastic modulus and higher ductility, making it particularly attractive for applications requiring greater strain accommodation.

The thermodynamic predictions and experimental validations together provide a comprehensive understanding of the alloys’ phase stability and thermal behavior. The consistent presence of the β phase above the β-transus temperature and the predictable phase transitions highlight the alloys’ suitability for advanced applications, including biomedical implants and high-temperature components. For biomedical applications, the ability to fine-tune the β-transus temperature through composition offers a pathway to optimize mechanical properties, such as elasticity and fatigue resistance, critical for load-bearing implants.

### Microstructural Characterization

Materials microstructure, which refers to the arrangement of atoms within the material, is of utmost importance for its properties, as it determines the characteristics the material will exhibit.^31^ Therefore, microstructural characterization is essential for understanding the properties displayed by materials, as well as for comprehending the processes that can alter the microstructure to achieve the desired properties.^19,31,39^

Table 3 presents the semi-quantitative chemical composition obtained through Energy Dispersive Spectroscopy (EDS), while the interstitial element, oxygen, was detected using a LECO RO-400 oxygen analyzer.

**Table 3:** Semiquantitative Chemical Analysis Obtained via SEM-EDS and Oxygen Content Determined using the LECO RO-400 Oxygen Analyzer for BCC Multiprincipal Alloys

alloys	alloying elements (wt %)				
	Ti	Nb	Zr	Ta	O
Ti–25Nb–25Zr–25Ta	balance	23.1	25.1	28.2	0.046
Ti–33Nb–33Zr	balance	33.4	34.0	--	0.099
Ti-40Nb-40Zr	balance	40.9	38.1	--	0.126

It can be observed from Table 3 that the chemical composition found is close to the nominal composition, considering that the technique used for its determination is semi-quantitative. Regarding oxygen, despite all precautions taken during alloy preparation, slight oxygen contamination occurred during processing, which was conducted outside of a protective atmosphere.

Figure 3 shows the X-ray diffraction patterns for the 25Ta, 33Zr, and 40Zr alloys in the HR condition, along with the respective diffraction planes. In general, for all the alloys presented, the β phase (2θ = (110); (200); (211); and (220)) is predominant in the HR condition, while the α″ phase (2θ = (200); (220); and (411)) is observed as a minor phase.

**Figure 3:**
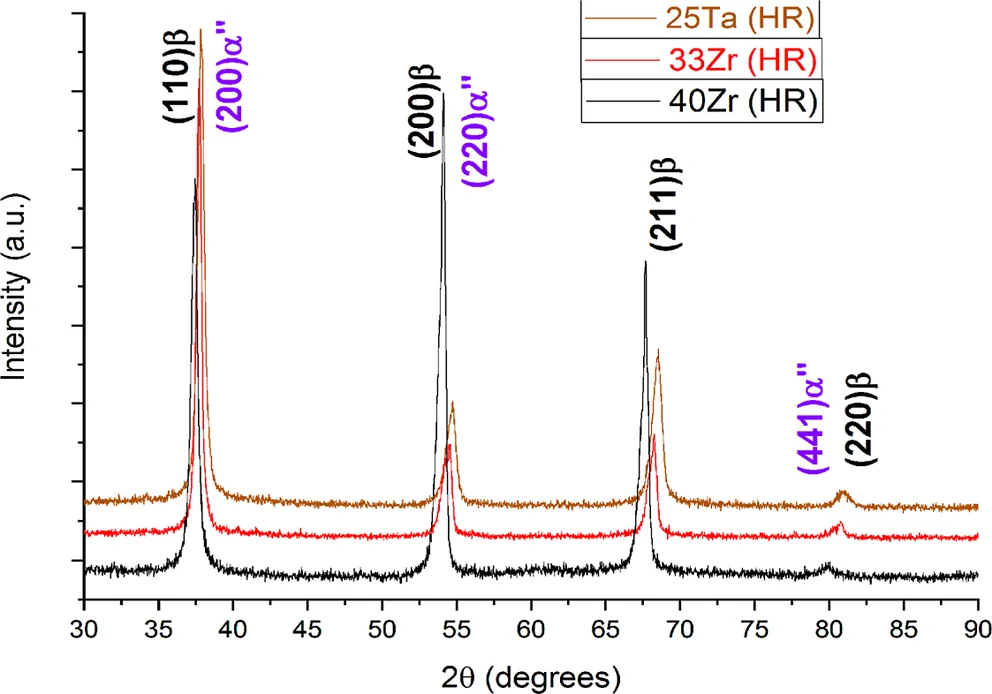
XRD Patterns in the hot rolled (HR) condition for the alloys: (a) 25Ta; (b) 33Zr; and (c) 40Zr.

Based on the thermomechanical history of the alloy, it is evident that the α’’ phase originated during the hot rolling process. This phase (α’’ - stress-induced martensite, SIM) emerged due to deformation, which is consistent with the findings of other authors.^4,10,19,38,40^ . Additionally, the literature indicates that alloys in the as-cast condition typically exhibit a dendritic structure.^16^ However, the microstructure undergoes a morphological transformation during hot rolling, transitioning from dendritic to equiaxed, as a result of dynamic recrystallization.^19,40^

Furthermore, Figure 3 shows that the β-phase diffraction peaks are shifted toward lower 2θ angles, with this effect being more pronounced for the 40Zr, 33Zr, and 25Ta alloys. This behavior is directly related to the influence of the alloying elements on the β-phase lattice parameter. Among the alloying elements investigated, Zr has the largest atomic radius (≈1.60 Å), whereas Ti, Nb, and Ta exhibit atomic radii of approximately 1.47 Å, 1.46 Å, and 1.46 Å, respectively. Therefore, increasing the Zr content promotes an expansion of the β-phase (BCC) crystal lattice, increasing both the lattice parameter and the interplanar spacing. According to Bragg’s law, this increase results in a systematic shift of the diffraction peaks toward lower 2θ angles. Consequently, the 40Zr alloy, containing the highest Zr content (40 at. %), exhibits the largest peak shift, followed by the 33Zr alloy (33 at. % Zr), whereas the 25Ta alloy, which contains only 25 at. % Zr with partial replacement by Ta, shows the smallest peak displacement. This behavior confirms that increasing the Zr content promotes the expansion of the β-phase crystal lattice, which is directly reflected in the position of the diffraction peaks.

Using X-ray diffractograms, the lattice parameter of the β phase in the studied alloys was calculated via Bragg’s law and the interplanar spacing for the cubic system.^16^ The calculated values are presented in Table 4 in the following subsection. The observed trends will be discussed alongside the mechanical properties in the next subsection.

**Table 4:** Values for Lattice Parameter for β Phase (a - Å), Elastic Modulus (E - GPa), Vickers Hardness (HV_0.5_), Average Grain Size (A.G.S. - μm), Entropy Variation (Δ*S* - J/K), β-transus Temperature (°C), and Density (g/cm^3^) for the Alloys 25Ta, 33Zr, and 40Zr Under HR Conditions, as Well as Data for the as-cast Condition Obtained From the Literature^36,38^

properties	25Ta	33Zr	40Zr
lattice parameter bcc,*a*(Å)	3.3613 ± 0.002	3.3608 ± 0.001	3.3963 ± 0.002
elastic modulus (GPa) – AC^a^	(73 ± 2)^38^	(79 ± 3)^40^	(63 ± 1)^40^
elastic modulus (GPa) - HR	59 ± 3	59 ± 1	42 ± 2
vickers microhardness (HV_0.5_) – AC^a^	(323 ± 7)^38^	(247 ± 14)^40^	(262 ± 17)^40^
vickers microhardness (HV_0.5_) - HR	316 ± 23	314 ± 23	293 ± 12
avarege grain size (μm)	113 ± 27	80 ± 14	73 ± 15
Δ*S*(J/K)	1.283	1.041	1.098
β-transus(°C)	490	471	462
density (g/cm^3^)	7.35	6.11	6.62

a Data from the literature.

Figure 4 presents the microstructures of three hot-rolled alloys, designated as 25Ta, 33Zr, and 40Zr, shown through optical microscopy (left) and scanning electron microscopy (SEM) (right). All alloys exhibit two primary phases: the β phase (matrix) and the α’’ phase (stress-induced martensite, SIM). The β phase serves as the matrix in all cases, forming large, equiaxed grains indicative of recrystallization during hot rolling. The α’’ phase appears as elongated or block-like regions distributed within the β matrix, resulting from stress-induced transformations during thermomechanical processing.^4,10,19,40^ Notably, the SEM images reveal clean grain boundaries and uniform microstructures across all alloys, reflecting the effectiveness of the hot-rolling process in enhancing microstructural integrity.

**Figure 4:**
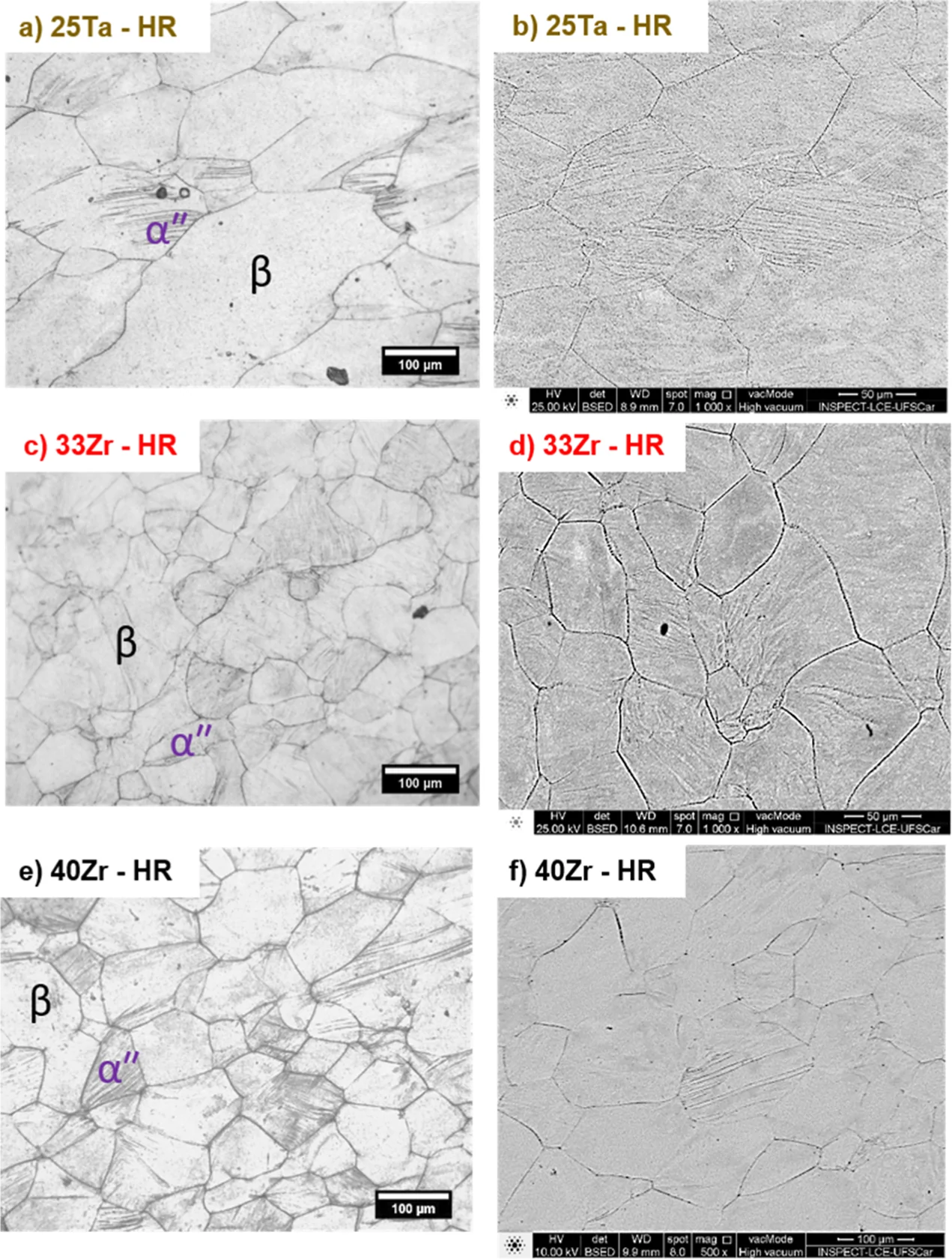
Optical microscopy images for 25Ta (b), 33Zr (d) and (f) 40Zr alloys and, scanning electron microscopy (SEM) images in BSED mode for the 25Ta (b), 33Zr (d) and (f) 40Zr alloys.

Distinct differences among the alloys highlight the influence of alloying elements on their microstructures. The 25Ta alloy displays large, equiaxed β grains with localized α’’ regions, while the 33Zr alloy exhibits a finer grain structure with elongated α’’ regions (observable in the central region of Figure 3d), suggesting increased recrystallization and residual stresses. The 40Zr alloy, characterized by smaller grains and a more homogeneous α’’ phase distribution, demonstrates a higher degree of dynamic recovery or extensive recrystallization. These observations underscore the significant role of Ta and Zr in governing grain size, recrystallization behavior, and the morphology of the α’’ phase.^41–43^

To better comprehend the microstructural behavior of the 25Ta, 33Zr, and 40Zr alloys in the hot-rolled (HR) condition, Transmission Electron Microscopy (TEM) analysis was conducted, as depicted in the bright field and selected area diffraction (SAD) patterns in Figure 5. These TEM images provide deeper insight into the phase distributions and crystallographic features of the alloys.

**Figure 5:**
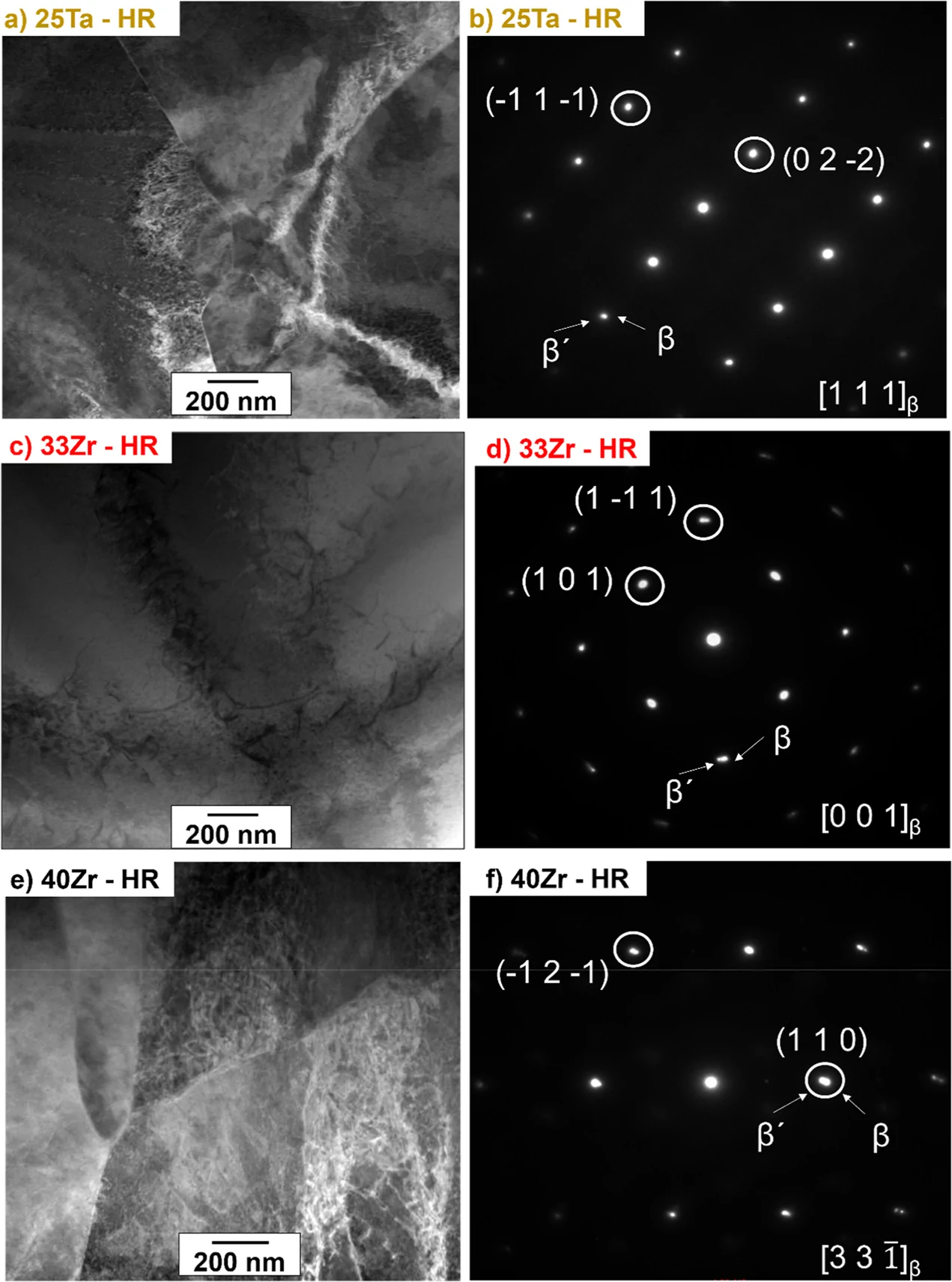
Transmission electron microscopy (TEM) images in bright field for the 25Ta (a) and 33Zr (c) alloys, and in dark field for the 40Zr alloy (e), along with their corresponding selected area electron diffraction (SAD) patterns.

The bright field images reveal a high degree of contrast between two distinct phases, the β and β′ phases, which exhibit differences in chemical composition. These compositional variations lead to differences in lattice parameters, which are responsible for the observed contrast.^26,38,44,45^ The presence of these two phases in the alloy microstructure suggests a phase separation phenomenon, possibly linked to the chemical instability of the β phase during thermomechanical processing.

Despite the prediction of a single β phase in the thermodynamic equilibrium diagrams generated using the ThermoCalc software, the TEM observations indicate the coexistence of β and β′ phases. This divergence arises from the fact that ThermoCalc assumes equilibrium-cooling conditions, while the samples analyzed here are in a non-equilibrium hot-rolled condition. The electron diffraction patterns (SAD) further confirm the decomposition of the β phase into β and β′ phases, which were identified based on the diffraction spots corresponding to specific crystallographic planes. The zone axes were indexed as [001] β and [-113] β, with corresponding reflections from these planes observed in the SAD patterns.

In the 25Ta alloy (Figure 5a,b), both the β and β′ phases were identified in the bright field image. The overlap of diffraction spots in the SAD pattern indicates the simultaneous presence of these two phases. This indicates that the β phase undergoes decomposition into a more chemically ordered β′ phase due to the thermomechanical history, specifically during hot rolling. This type of downhill decomposition (where β decomposes into β and β′) has been reported in the literature and is characteristic of alloys with high β -stabilizing element content, such as tantalum and zirconium.^26,38,44,45^

The 33Zr and 40Zr alloys (Figure 5c,f) exhibit similar behavior, with the β phase being dominant and showing evidence of β′ formation. The diffraction patterns reveal reflections corresponding to the same crystallographic planes as those seen in the 25Ta alloy, further confirming the presence of the β and β′ phases.^26,38,44,45^ The decomposition process in these alloys is likely influenced by their high Zr content, which acts as a strong β stabilizer, promoting the formation of the β′ phase during hot rolling.

A closer examination of Figure 5f reveals the presence of diffuse regions in the image. By adjusting the image brightness, the diffuse scattering becomes more clearly visible, as shown in Figure 6.

**Figure 6:**
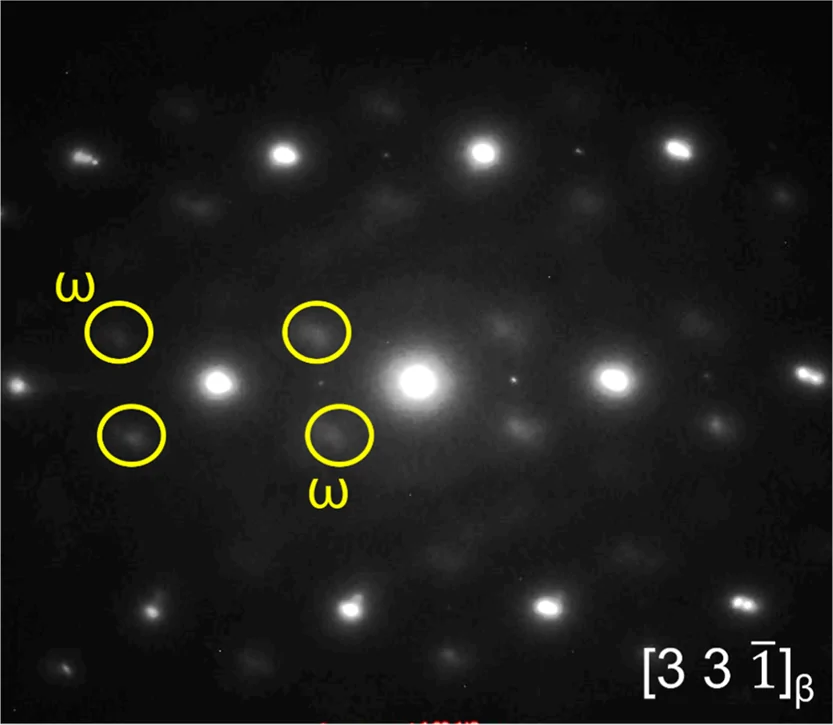
Enhanced contrast image obtained from Figure 5f (SAD for 40Zr-HR), highlighting the presence of diffuse scattering regions. The adjustment in image brightness allows for clearer visualization of these features.

Diffuse scattering is observed in a distinct region. In this region, outside the line of β and β′ reflections (highlighted in yellow), spots associated with the ω phase are identified, as reported in the literature.^46^ The ω phase is a metastable phase frequently observed in β-Ti alloys during cooling processes or thermal treatments and may act as an important strengthening mechanism due to its interaction with the β matrix. The precipitation of the ω phase generally promotes an increase in strength and hardness by hindering dislocation motion; however, when present in a high volume fraction or continuous distribution, this phase may decrease ductility and increase the elastic modulus due to its higher stiffness compared with the β phase.

These findings suggest that, although the ω phase is commonly considered to vanish under specific conditions, as high content of β-stabilizer elements, it may, in fact, persist, and most likely does, even in extremely small amounts in β alloys.^19,46,47^ Therefore, while Zr is known to suppress the ω phase, it does not fully eliminate it from the microstructure, in contrast to what is reported in the literature.^48–52^

### Physical and Mechanical Characterization

Table 4 presents the values for lattice parameter (a - Å), elastic modulus (E - GPa), Vickers hardness (HV_0.5_), average grain size (A.G.S. - μm), entropy variation (Δ*S* - J/K), β-transus temperature (°C), and density (g/cm3) for the alloys 25Ta, 33Zr, and 40Zr under HR conditions. Some data were obtained from the literature and included in the table in order to enable a comparison between the results observed in the HR condition in the present study and those reported for the AC condition in other studies.

When considering only the elastic modulus, the 40Zr alloy stands out as a strong candidate for biomedical applications due to its low modulus (42 GPa), which is closer to human bone, thereby reducing the risk of stress shielding.^10,11^ Although the ω phase was detected in the 40Zr alloy by TEM analysis, this alloy still exhibited a very low elastic modulus value. This indicates that the ω phase is present in a small fraction and finely dispersed within the β matrix, without a significant contribution to the overall elastic response of the material.

In Table 4, it is possible to observe a discrepancy between the elastic modulus values reported for β-Ti alloys in previously published conditions (as-cast) and those obtained for the alloys produced in this work after hot-rolling (HR) processing. This difference is mainly related to the influence of the processing route on the microstructure and phase stability. β-Ti alloys in the as-cast condition generally exhibit higher elastic modulus values due to their initial microstructural state, whereas hot-rolling thermomechanical processing promotes significant microstructural modifications, such as grain refinement, changes in crystallographic texture, and stabilization of the metastable β phase. These modifications can reduce the elastic stiffness of the material and consequently result in lower elastic modulus values. Therefore, the reduced elastic modulus values observed for the HR alloys investigated in this study can be attributed to the combined effects of the alloy composition, the predominantly β-phase microstructure, and the modifications induced by thermomechanical processing. This behavior has been previously reported for β-Ti alloys, in which hot-rolled conditions exhibited lower elastic modulus values compared with the corresponding as-cast conditions, demonstrating that the processing route plays a fundamental role in controlling the elastic properties of these alloys.^53^

The elastic moduli of the hot-rolled (HR) alloys were significantly lower than those of conventional metallic biomaterials used for orthopedic implants. The 25Ta and 33Zr alloys exhibited elastic moduli of 59 ± 3 GPa and 59 ± 1 GPa, respectively, whereas the 40Zr alloy showed an even lower value of 42 ± 2 GPa. In comparison, commercially pure titanium (CP–Ti) and Ti–6Al-4 V exhibit elastic moduli of approximately 102–105 GPa and 110–114 GPa, respectively. β-type titanium alloys, such as Ti–10Mo, generally present elastic moduli in the range of 60–75 GPa, while Ni–Ti alloys exhibit values between approximately 30 and 83 GPa, depending on their phase constitution and thermomechanical processing. Therefore, the alloys developed in the present study, particularly the 40Zr alloy, exhibit elastic moduli closer to that of cortical bone (10–30 GPa), thereby reducing the mechanical mismatch at the bone–implant interface.^10^ Although these values remain higher than those of cortical bone and, consequently, cannot completely eliminate the stress-shielding effect under physiological loading conditions, they represent a substantial improvement over conventional titanium alloys and are expected to mitigate stress shielding.

Regarding mechanical properties, significant variations in Vickers microhardness were observed among the alloys. Despite the typical reduction in hardness associated with the β → α″ phase transformation, both 33Zr and 40Zr alloys exhibited increased hardness values after thermomechanical processing.^40^ The increase in hardness results from strain hardening associated with a high dislocation density, combined with the chemical decomposition of the β phase into β + β′, which generates nanometric compositional fluctuations capable of hindering dislocation motion.^40^ For instance, the 40Zr alloy exhibited a microhardness of 293 HV_0.5_, the lowest among the three analyzed alloys, but it still demonstrates sufficient mechanical strength for biomedical applications. In contrast, the 25Ta alloy maintained its hardness (316 HV_0.5_) practically unchanged, indicating higher stability under thermomechanical processing.^19,31,44,54^

Structural and thermal differences between the alloys are also noteworthy. The analysis of lattice parameters for the body-centered cubic (BCC) structure revealed that the 40Zr alloy has the largest lattice parameter (3.3963 Å), which may have implications for its phase stability and mechanical behavior. Moreover, the 40Zr alloy exhibits the lowest β-transus temperature (462 °C), which facilitates its transformation during heat treatments, enabling the tuning of its mechanical and microstructural properties.

In terms of density, the 40Zr alloy is the second lightest (6.62 g/cm^3^), representing an advantage in biomedical applications where low-density materials are preferred to reduce implant weight. On the other hand, the 25Ta alloy, despite having the highest density (7.35 g/cm^3^), offers superior hardness and thermal stability, which might be advantageous for other specific applications.

Finally, the configurational entropy (Δ*S*) analysis revealed relatively similar values among the alloys, with the 40Zr alloy exhibiting a Δ*S* of 1.098 J/K. Although configurational entropy is a useful parameter for understanding phase stability and alloy design, the investigated alloys cannot be classified as high-entropy alloys (HEAs), since they do not satisfy the widely accepted criterion of containing at least five principal elements in near-equiatomic proportions. Instead, they should be regarded as β-type titanium alloys. The relatively high configurational entropy, combined with the microstructure characterized by an average grain size of 73 ± 15 μm, contributes to the balanced combination of mechanical and thermal properties observed in the 40Zr alloy, further reinforcing its potential for biomedical applications.

Figure 7 provides a graphical representation of the data outlined in Table 4.

**Figure 7:**
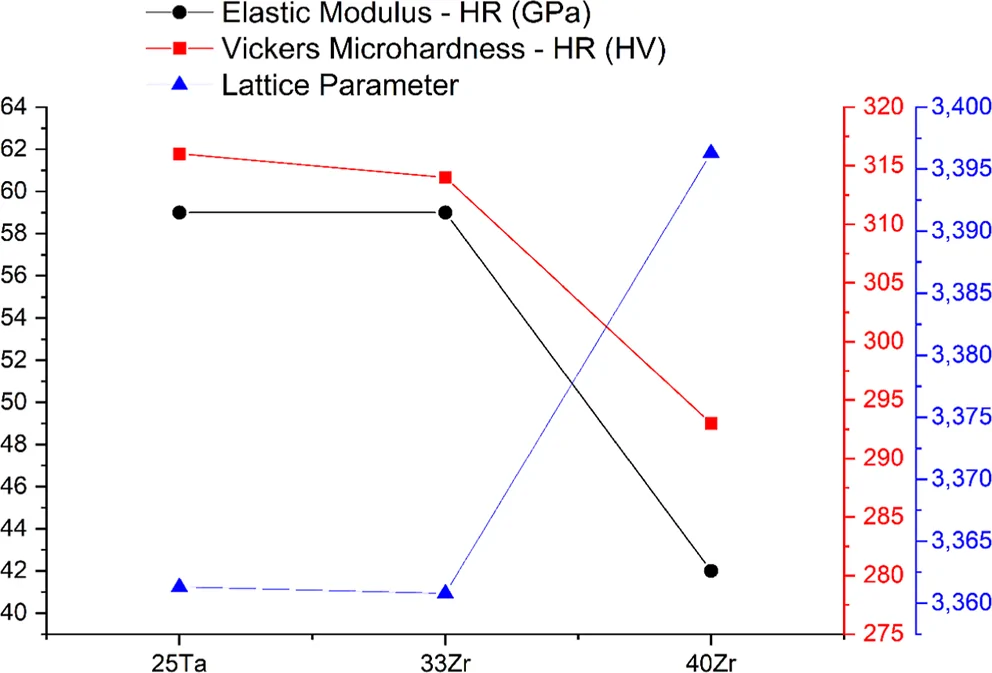
Comparative graph of the values of elastic modulus (E, GPa), Vickers microhardness (HV_0.5_), and lattice parameter (Å) for the alloys in HR condition: 25Ta, 33Zr, and 40Zr.

Figure 8 presents a graphical representation of the tensile test, showing an average obtained from six test specimens for a specific sample of each alloy. Meanwhile, Table 5 provides values for alloys found in the literature as well as the average values from the six tests conducted for each of the studied alloys (33Zr, 40Zr, and 25Ta).

**Figure 8:**
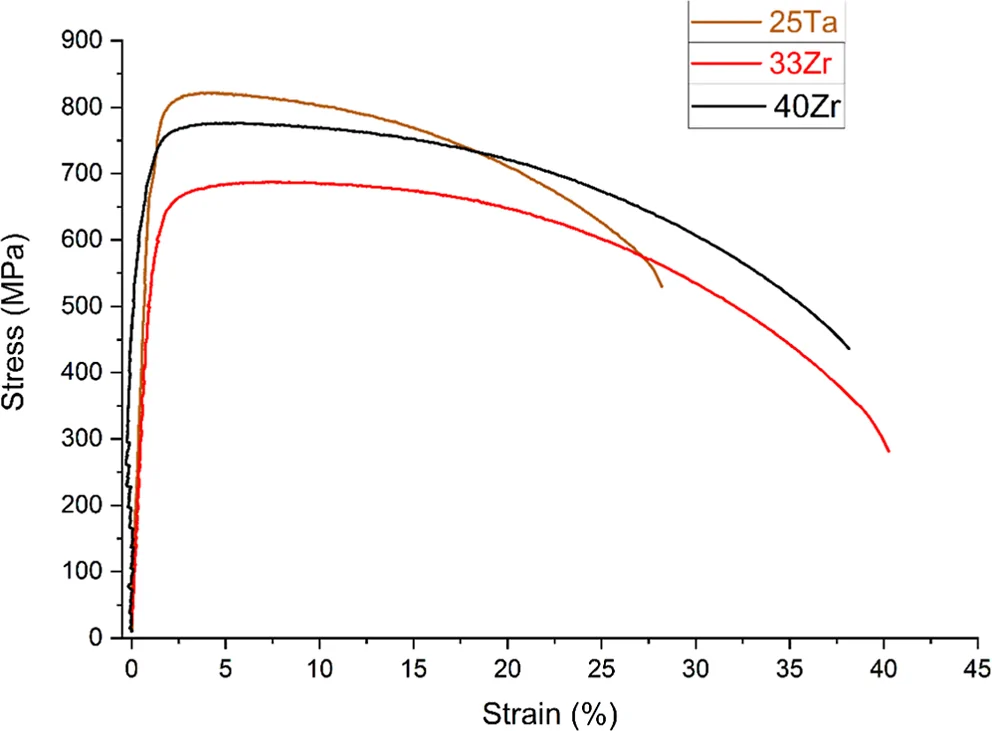
Representative tensile stress–strain curves of samples under HR conditions for the alloys: 25Ta, 33Zr, and 40Zr.

**Table 5:** Values Obtained from the Tensile Tests for the 25Ta, 33Zr, and 40Zr Alloys, in Hr Condition, Including Yield Strength (σ_*y*_), Ultimate Tensile Strength (σ_max_), Elongation (e), and Elastic Modulus (E)

alloy	σ_*y*_ (MPa)	σ_max_ (MPa)	e (%)	E (GPa)	Ref
25Ta - HR	536 ± 12	837 ± 44	27 ± 5	70 ± 27	present
33Zr - HR	424 ± 8	681 ± 16	42 ± 2	57 ± 6	present
40Zr - HR	517 ± 6	791 ± 10	39 ± 3	76 ± 14	present
c.p. Ti (Grade 4)	485	550	15	104	^57^
Ti–6Al–4V (wt %)	880	950	14	110–140	^57^
Ti–13Nb–13Zr (wt %)	555	733	19	84	^58^
Ti–16Nb–10Zr (wt %)	485	520	22	70	^59^
Ti–28Nb–35.4Zr (wt %)	729	755	11	65	^60^
Ti–22.5Nb–2Zr–0.7Ta^a^ (at. %)	---	537	16	56	^56^
Ti–22.5Nb–2Zr–0.7Ta–2O^a^ (at. %)	---	883	2.5	90	^56^

a Only these alloys are presented in (at. %), all the alloys are presented in (wt %).

Among the alloys studied, all exhibited lower elastic modulus and greater fracture elongation when compared to the Ti–6Al-4 V alloy, which is one of the most widely used alloys for implants today.^55^ While the reduction in elastic modulus is important as it mitigates the issue of stress shielding, it is crucial for the alloy to possess high mechanical strength, as other properties, such as fatigue resistance, are necessary for use as an implant. In this regard, the 25Ta alloy proved to be more promising, as it exhibits yield strength and ultimate tensile strength values that are approximately 61% and 88%, respectively, closer to those reported for the Ti64 alloy.

The quaternary alloy Ti-22.5Nb–2Zr-0.7Ta–2O (at.%) shows a ultimate tensile strength (σ_max_) higher than that of the 25Ta quaternary alloy; however, its elastic modulus is higher (90 GPa).^56^ Since the elastic modulus depends on the nature of atomic bonding and is highly sensitive to the present phase/structure, the variation in microstructure (phases) due to the presence of oxygen—a stabilizing element for the α phase—significantly alters the mechanical properties.^56^ This alteration is so pronounced that it can be observed through the type of fracture exhibited by the alloys during tensile deformation tests. For example, in the Ti-22.5Nb-0.7Zr–2Ta-xO alloys with x = 0, 0.5, 1, 1.5, and 2, the fracture is considered ductile up to 1.5% oxygen (at.%), while above these values, it is deemed brittle.^56^ This is evident when comparing the properties presented in Table 4.11.^56^ Therefore, it is essential to control the oxygen content in the alloys, as it can significantly alter the microstructure and, consequently, the properties of β-Ti alloys.

Comparing the average values of the elastic modulus determined by tensile testing (TT) with those obtained via Sonelastic (So), we observe that, in the HR condition for the 33Zr alloy, the values are 59 ± 1 GPa (So) and 57 ± 7 GPa (TT), which are very close and consistent with each other. However, for the 40Zr alloy in the HR condition, the values are 42 ± 2 GPa (So) and 76 ± 27 GPa (TT), while for the 25Ta alloy, the values are 59 ± 3 GPa (So) and 70 ± 14 GPa (TT). It is important to observe that the curves presented in this study (Figure 8) indicate that the elastic modulus are practically the same as those obtained via Sonelastic; thus, this measurement may not be far from reality. The discrepancy observed between these values may be associated with the non-homogeneity of the samples (as the values are highly sensitive to specimen preparation) and, consequently, the tensile test presenting a large variance in results, as evidenced by the associated measurement errors. This suggests the need for a larger number of tests to achieve a lower variation in the values observed. Furthermore, it is worth observing that the measurement obtained via Sonelastic is generally more reliable, as the associated error does not exhibit the significant variations seen in tensile testing, which can show variations greater than 50% between measurements.

Figure 9 presents SEM images (in SE mode) of the fracture surfaces of tensile test specimens for the 25Ta, 33Zr, and 40Zr alloys in the hot-rolled (HR) condition, offering insights into their fracture mechanisms under uniaxial tensile loading. All alloys exhibit dimples on their fracture surfaces, a characteristic feature of ductile fracture, confirming significant plastic deformation before rupture. This mechanism involves the nucleation, growth, and coalescence of microvoids, allowing the material to absorb substantial energy during deformation.^61^ The 25Ta alloy displays a dense, uniform distribution of dimples, reflecting homogeneous plastic deformation and effective stress transfer, facilitated by its higher hardness (316 HV_0.5_) and β-transus temperature (490 °C). In contrast, the 33Zr alloy shows a more heterogeneous fracture surface, with localized deformation features and less uniform dimple distribution, likely due to microstructural heterogeneities such as grain size variations or localized phase transformations.^57^ The 40Zr alloy exhibits a combination of larger dimples, indicative of substantial plastic deformation, and smoother regions, potentially associated with strain hardening or phase transformations, highlighting its balance of ductility and energy absorption.

**Figure 9:**
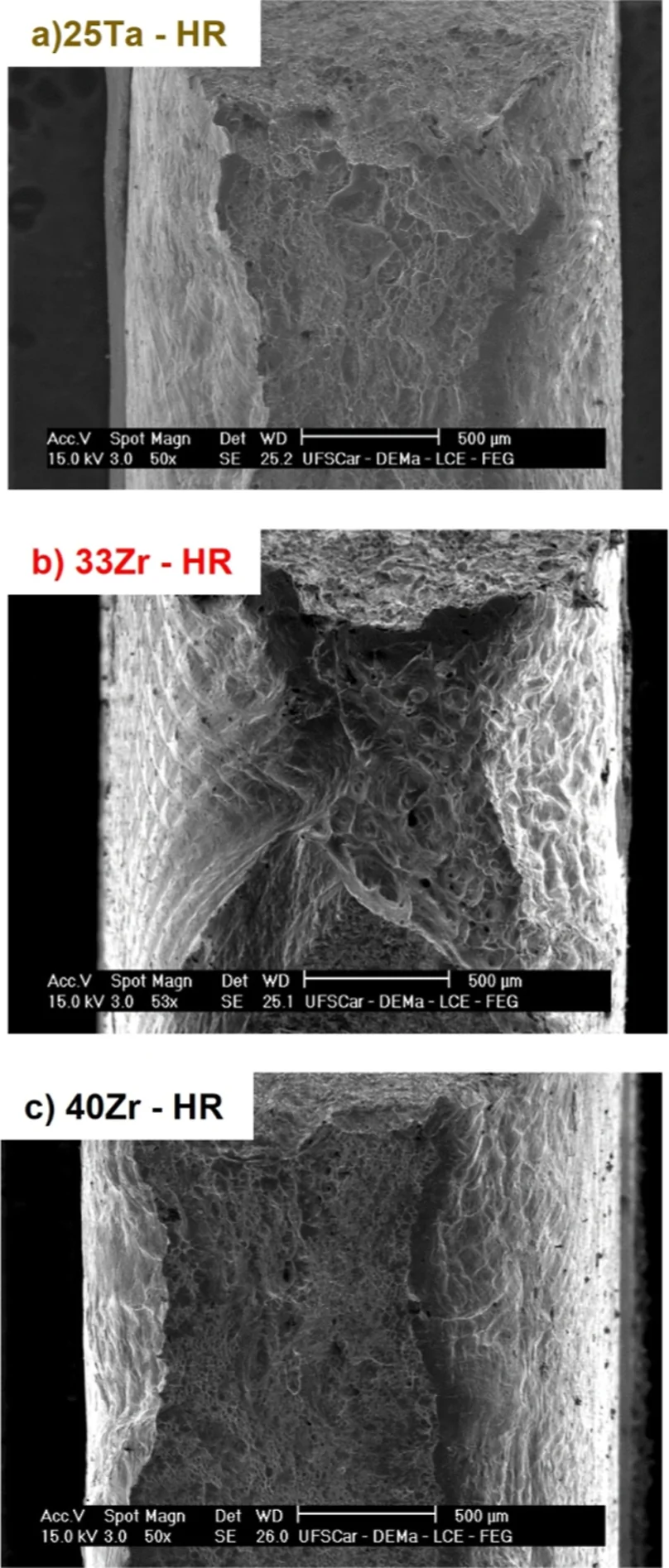
SEM image of the fracture surface of the test specimens used in the tensile test for the samples under HR condition: (a) 25Ta, (b) 33Zr, and (c) 40Zr.

These fracture surface characteristics underscore the predominantly ductile fracture mechanisms in all three alloys, with variations in dimple size and distribution reflecting the influence of composition, microstructural stability, and processing history. The 25Ta alloy demonstrates the most homogeneous deformation, aligning with its higher density and stability, while the 33Zr alloy reveals localized heterogeneities, and the 40Zr alloy combines high plasticity with enhanced strain accommodation due to its lower elastic modulus (42 GPa). These findings emphasize the critical role of microstructural control in tailoring mechanical properties for specific applications, such as biomedical implants, where toughness and ductility are essential.^62^ The observed energy-absorbing mechanisms highlight the suitability of these alloys for load-bearing applications, aligning with prior studies on ductile fracture behavior governed by dimples and microvoid.^57,61^

### Electrochemical Characterization

The electrochemical behavior of the 25Ta, 33Zr, and 40Zr alloys was investigated in Simulated Body Fluid (SBF) at 36.5 °C to evaluate their corrosion resistance under physiological conditions. Figure 10 presents the variation of the open-circuit potential (E_oc_) during 1 h of immersion for both as-cast (AC) and hot-rolled (HR) conditions.

**Figure 10:**
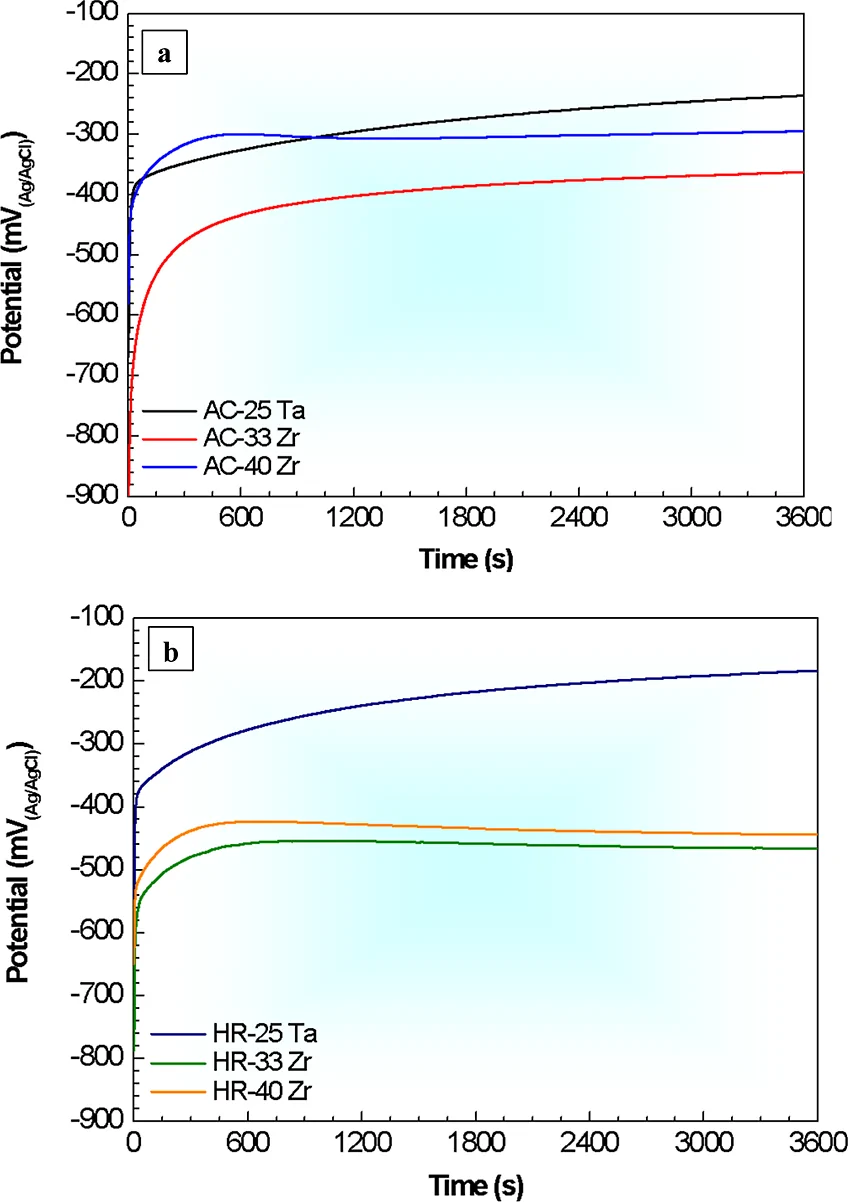
Variation of the E_oc_ over 1 h of immersion in SBF at 36.5 °C for the 25Ta, 33Zr, and 40Zr samples: (a) AC, and (b) HR conditions.

All alloys exhibited a rapid stabilization of E_oc_ values within the first few minutes of immersion, indicating the spontaneous formation of a protective oxide film on the surface. In the AC condition, the 25Ta alloy showed the most noble potential, stabilizing at approximately −250 mV_Ag/AgCl_, suggesting the development of a more stable and protective passive layer. Conversely, the 33Zr alloy exhibited the lowest potential values, around −380 mV_Ag/AgCl_, revealing a comparatively less stable passive film throughout the immersion period. The 40Zr alloy showed an intermediate behavior, with Eoc value close to −300 mV_Ag/AgCl_.

In the HR condition, a distinct trend was observed between the Ta- and Zr-containing alloys. The 25Ta alloy exhibited a shift toward more positive potentials, stabilizing at approximately −180 mV_Ag/AgCl_, indicating an improvement in the stability of the passive film after hot rolling. In contrast, the 40Zr and 33Zr alloys presented more negative potentials, around −440 mV_Ag/AgCl_ and −480 mV_Ag/AgCl_, respectively, suggesting a slight decrease in the thermodynamic stability of the oxide film compared to their as-cast counterparts. This difference may be associated with compositional and structural factors: the presence of Ta promotes the formation of highly stable Ta_2_O_5_, whereas in Zr-rich alloys, the oxide film – mainly composed of ZrO_2_ and Nb_2_O_5_ – may be thinner or less compact immediately after processing. Despite these variations, all alloys exhibited stable potentials over time, confirming their spontaneous passivation in SBF solution. Additionally, the E_oc_ values for both conditions of the 33Zr and 40Zr alloys are consistent with the formation of mixed TiO_2_–Nb_2_O_5_–ZrO_2_ surface oxides, which are typical of Ti–Nb–Zr systems in this kind of electrolyte.^63,64^

The potentiodynamic polarization curves presented in Figure 11 corroborate the trends observed in the E_oc_ measurements. All alloys displayed passive behavior characterized by very low corrosion current densities (i_corr_) – on the order of 10^–7^ A/cm^2^ - and extended passive regions that remained stable up to 3000 mV_Ag/AgCl_, a value much higher than any rest potential recorded in vivo for metallic biomaterials.^65^ The corresponding electrochemical parameters are summarized in Table 6, which lists the values extracted from the polarization curves obtained in SBF solution for the 25Ta, 33Zr, and 40Zr samples in both AC and HR conditions.

**Figure 11:**
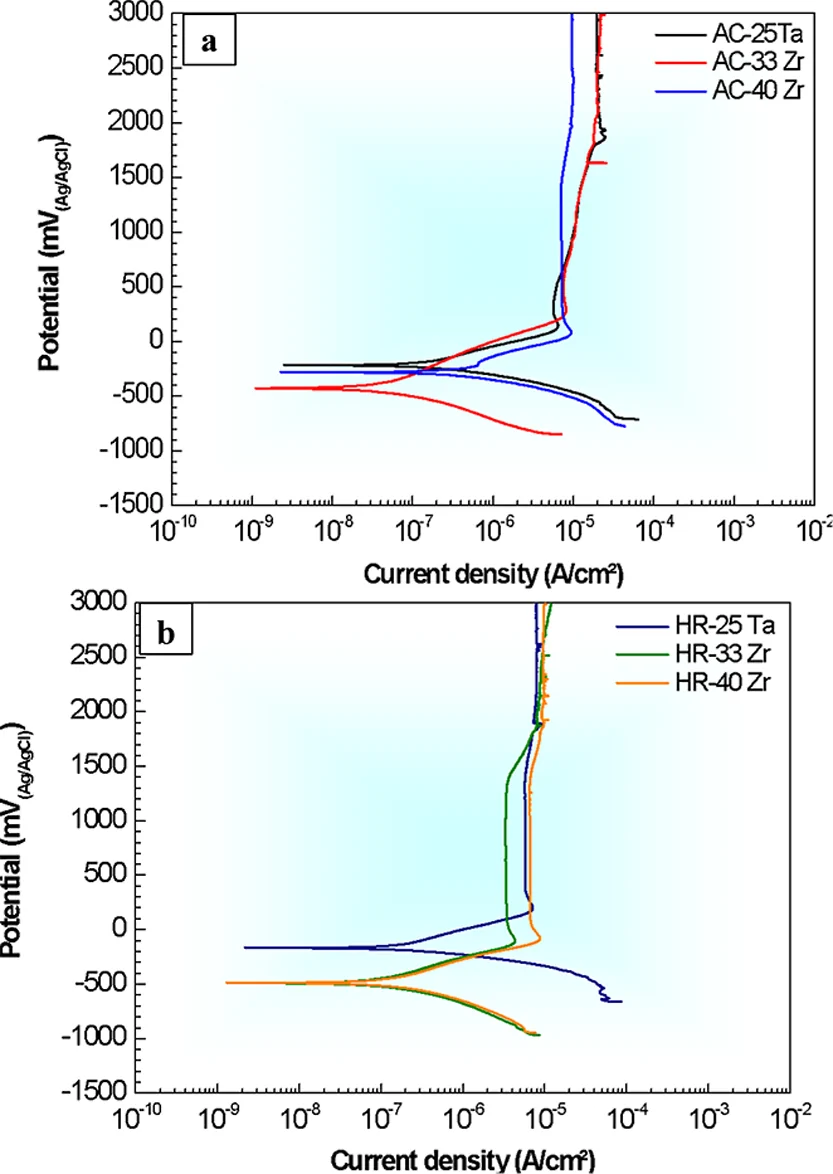
Potentiodynamic polarization curves in SBF solution for the 25Ta, 33Zr, and 40Zr samples: (a) AC, and (b) HR conditions.

**Table 6:** Average electrochemical Parameters Obtained From the Potentiodynamic Polarization Curves in SBF Solution for the 25Ta, 33Zr, and 40Zr Alloys in the as-cast (AC) and Hot-Rolled (HR) Conditions

samples	E_corr_ (mV_SCE_)	i_corr_ (μA/cm^2^)	i_pass_ ^a^ (μA/cm^2^)	corrosion rate – CR^b^ (10^–2^mpy)
AC-25Ta	–274 ± 31	0.31 ± 0.04	6.30 ± 0.31	8.36 ± 0.16
AC-33Zr	–330 ± 52	0.27 ± 0.01	6.99 ± 0.05	8.93 ± 0.04
AC-40Zr	–414 ± 35	0.26 ± 0.03	8.44 ± 0.10	8.68 ± 0.09
HR-25 Ta	–184 ± 12	0.29 ± 0.07	6.82 ± 0.83	8.04 ± 0.23
HR-33 Zr	–452 ± 64	0.26 ± 0.02	7.61 ± 0.07	8.78 ± 0.05
HR-40 Zr	–481 ± 28	0.26 ± 0.09	7.54 ± 0.10	8.70 ± 0.02

a Current density values taken at 1000 mV_Ag/AgCl_.

b Values estimated according to ASTM G 102 standard.

Both conditions of 25Ta alloy showed the most favorable electrochemical response, with the highest corrosion potential (E_corr_) and the lowest corrosion rate (CR) and passive current density (i_pass_) values, confirming its superior corrosion resistance. This performance is primarily attributed to the presence of Ta_2_O_5_ within the surface oxide layer mainly composed of TiO_2_, which is known to be chemically stable and highly insulating, effectively suppressing ionic transport across the metal/solution interface.^65–68^

The 33Zr and 40Zr alloys also exhibited passive behavior, though with slightly higher passive current densities and less noble potentials. These differences can be related to the predominance of ZrO_2_ in their surface films, which, although chemically stable, presents higher ionic conductivity than Ta_2_O_5_, leading to a less compact and slightly more defective passive layer.^68,69^ Despite these differences, all alloys demonstrated E_corr_ values more noble or comparable to those typically reported for wrought Ti–6Al–4 V in SBF, together with lower i_corr_, confirming the excellent corrosion resistance of the designed compositions.^70,71^

When comparing the two processing conditions in terms of the average electrochemical parameters, the HR samples tended to exhibit slightly lower i_pass_ values and broader passive regions than the AC ones. This improvement can be attributed to the improved microstructural uniformity and chemical homogeneity achieved after hot rolling, favoring the formation of a more compact, uniform, and adherent oxide film. These findings highlight the beneficial effect of thermomechanical processing on the corrosion resistance of β-Ti alloys.

The electrochemical impedance spectroscopy (EIS) outcomes illustrated in Figures 12 and 13 provide complementary information about the nature of the passive films and the corrosion behavior of the alloys. The Nyquist diagrams (Figure 12) for both the as-cast (AC) and hot-rolled (HR) conditions display a single capacitive loop, typical of passive systems controlled by charge-transfer reactions across a dense oxide layer. This similar impedance response indicates that identical corrosion mechanisms prevail on the surfaces of the different alloys.

**Figure 12:**
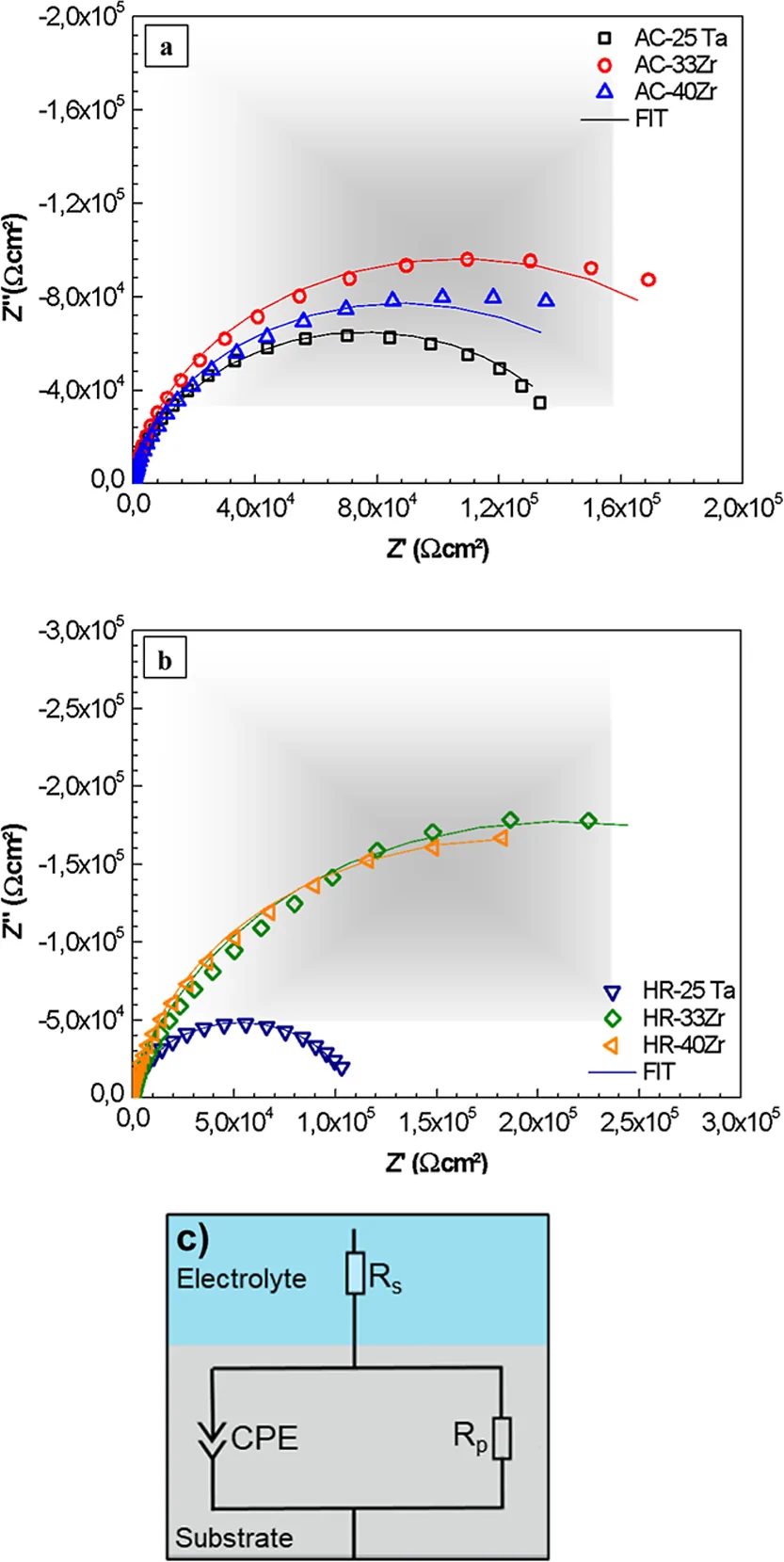
Nyquist plots obtained in SBF solution for the 25Ta, 33Zr, and 40Zr samples: (a) AC, (b) HR conditions and (c) EEC used for fitting the EIS experimental data for the 25Ta, 33Zr, and 40Zr samples in a SBF solution.

**Figure 13:**
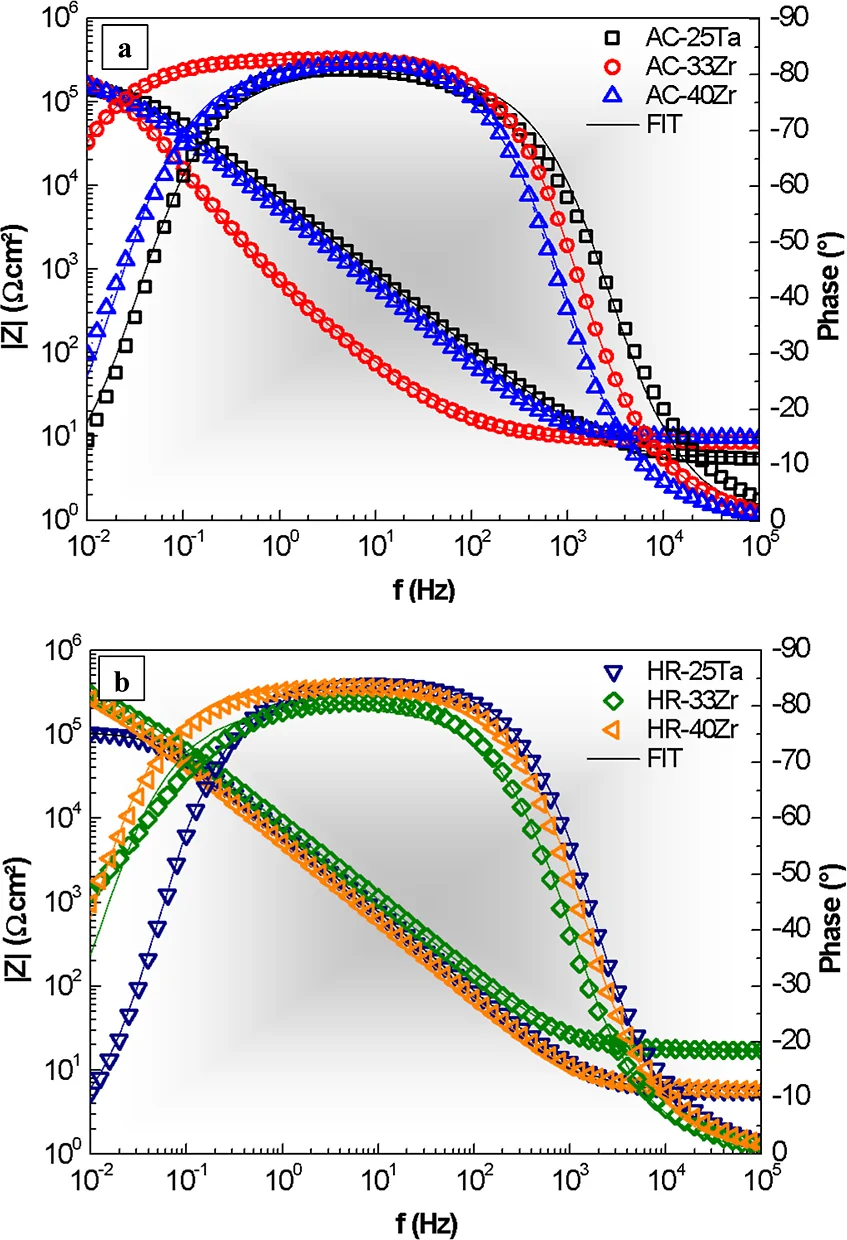
Bode plots obtained in SBF solution for the 25Ta, 33Zr, and 40Zr samples: (a) AC, and (b) HR conditions.

The semicircle diameters, which are directly related to the polarization resistance (R_p_) and inversely to the corrosion current density (i_corr_), show that the alloys exhibit comparable passive responses, with the highest R_p_ generally observed for 33Zr and the lowest for 25Ta. Moreover, the HR specimens exhibit larger semicircle diameters compared with their AC counterparts, reflecting higher R_p_ values and improved corrosion resistance. This improvement can be attributed to microstructural refinement and enhanced chemical uniformity induced by hot rolling, which facilitate the development of a more compact and adherent oxide film.

It should be borne in mind that although R_p_ is inversely related to i_corr_ through the Stern–Geary equation (i_corr_ = B/R_p_), the proportionality constant B depends on the anodic and cathodic Tafel slopes (β_a_ and βc) and may vary between alloys and processing conditions. Furthermore, for passive Ti-based systems in SBF, the Tafel regions are narrow and sometimes ill-defined, leading to some degree of uncertainty in i_corr_ obtained from polarization curves compared to R_p_ values derived from EIS at E_oc_. These factors help to explain minor differences in the ranking of corrosion resistance when comparing polarization and impedance results, without contradicting the overall observed trends.

In agreement with these findings, the Bode and phase-angle plots shown in Figure 13 confirm these trends. All alloys display phase angles approaching −80° across a wide frequency domain, indicative of a nearly ideal capacitive behavior typical of stable passive layers. Additionally, the HR samples present slightly higher impedance modulus (|Z|) values at low frequencies, further supporting the conclusion that hot rolling enhances the protective quality of the oxide film.

It is well established that the metal-electrolyte interface involved in electrochemical reactions can be represented by an impedance response analogous to that of an electronic circuit consisting of resistive and capacitive components.^72^ Hence, to deepen the impedance analysis and provide quantitative interpretation of the EIS results obtained for the investigated β-Ti alloys, the experimental data were fitted using equivalent electrical circuits (EEC). The choice of the most representative circuit was guided by previous studies and three main criteria: (i) the model should include the smallest possible number of elements; (ii) all elements must have a clear physical meaning in relation to the system analyzed; and (iii) the fit quality should be satisfactory, with χ^2^ ≤ 10^–3^ and individual parameter errors below 5%.^72^

The circuit illustrated in Figure 12c – represented as R_s_ (CPE­(R_p_)) – met all these criteria and was adopted for data fitting. It consists of a solution resistance (R_s_) in series with a parallel arrangement of a constant phase element (CPE) and a polarization resistance (R_p_). The physical significance of each element is as follows: R_s_ corresponds to the resistance of the electrolyte, CPE accounts for the non-ideal capacitive behavior associated with the oxide layer, and R_p_ reflects the charge-transfer resistance controlling the corrosion process at the metal/solution interface.^73–75^ It should be mentioned that alternative two-time-constant circuits were also tested but did not yield a meaningful improvement in fit quality, confirming that the single-time-constant model adequately represents the electrochemical response.

The adoption of a CPE instead of an ideal capacitor was required to account for the frequency dispersion typically observed in real electrochemical systems. Such dispersion originates from surface heterogeneities – including roughness and porosity – as well as from non-uniform current distribution across the electrode. The impedance of a CPE (Z_CPE_) is expressed as

1 $$Z_{CPE}={\left[Y_{0}{\left(j\omega \right)}^{\alpha }\right]}^{-1}$$

where Y_0_ is a parameter related to the capacitance (with dimensions of S.s^α^/cm^2^); j is the imaginary unit (j^2^ = −1); ω is the angular frequency, and α is an empirical exponent that quantifies the deviation from ideal capacitive behavior. The CPE behaves as an ideal capacitor when α = 1, exhibits a diffusion-controlled (Warburg-type) response when α = 0.5, corresponds to a pure resistor for α = 0, and mimics a pure inductor when α = −1.^76,77^

The impedance parameters summarized in Table 7 provide complementary insight into the corrosion behavior of the investigated alloys. The low χ^2^ values (10^–3^ range) confirm the reliability of the fitting procedure and the suitability of the selected equivalent circuit to describe the system. The solution resistance (R_s_) remained nearly constant for all specimens – between approximately 5.6 and 6.6 Ω.cm^2^ – indicating similar electrolyte conductivity and confirming that differences in impedance response arise mainly from the characteristics of the passive films.

**Table 7:** Average Impedance Parameters Determined in SBF Solution at the E_oc_ for the 25Ta, 33Zr, and 40Zr Alloys in the as-cast (AC) and Hot-Rolled (HR) Conditions

sample	Rs (Ω.cm^2^)	R_p_ (kΩ.cm^2^)	CPE*		Χ^2^ (10^–3^)
			Q (μS.s^α^/cm^2^)	α	
AC-25Ta	5.88 ± 0.18	127.92 ± 18.97	33.06 ± 2.44	0.92 ± 0.094	1.17
AC-33Zr	6.04 ± 0.19	389.33 ± 46.39	36.79 ± 6.29	0.92 ± 0.052	1.58
AC-40Zr	5.97 ± 0.16	284.42 ± 11.69	37.76 ± 5.35	0.91 ± 0.013	1.94
HR-25 Ta	5.60 ± 0.29	96.13 ± 16.97	31.95 ± 4.57	0.93 ± 0.073	0.59
HR-33 Zr	6.64 ± 0.22	157.81 ± 25.24	38.85 ± 3.49	0.89 ± 0.021	0.56
HR-40 Zr	6.11 ± 0.47	339.45 ± 40.06	41.82 ± 5.61	0.92 ± 0.011	1.16

The polarization resistance (R_p_) values are comparatively high for all samples, confirming the passive character and low charge-transfer rates of the β-Ti alloys in SBF. Although some variations are observed between compositions and processing conditions, R_p_ generally remains in the 10^2^ – 10^3^ kΩ.cm^2^ range, which is typical of stable passive Ti-based systems.^73–75^ The slight variations in R_p_ among the different samples suggest subtle modifications in film structure – possibly related to changes in oxide thickness or local composition – without implying any degradation of the overall protective behavior. Moreover, the α exponents, close to unity, together with the similar Q values (30 – 40 μS·s^α^/cm^2^), confirm a highly capacitive response and indicate oxide films of comparable thickness and dielectric properties, consistent with compact and adherent passive layers typically observed for β-Ti alloys. Altogether, these results indicate that all alloys develop robust passive films that effectively impede ionic transport across the metal/solution interface, in agreement with the trends previously observed from polarization and potential measurements.

In microstructural terms, the set of results demonstrates that thermomechanical processing by hot rolling (HR) acts synergistically on the microstructure and, consequently, on the mechanical and electrochemical properties of the developed alloys. The dynamic recrystallization promoted by HR eliminates the dendritic structure typical of the as-cast state (AC), refines the grains and homogenizes the distribution of β and α″ phases, which contributes to the reduction of the elastic modulus and the increase in hardness, due to work hardening and the β → β + β′ transition. This microstructural refinement also favors the formation of more compact and adherent passive films, as evidenced by the higher polarization resistance values, as well as lower passivation current densities in the HR samples. The presence of secondary phases, such as α′′ and/or ω, occurs in reduced fractions and is finely distributed, without significantly compromising the low modulus of elasticity or the excellent corrosion resistance observed. Thus, the combination of appropriate chemical composition and thermomechanical processing proves essential to balance mechanical strength and electrochemical stability, reinforcing the potential of beta alloys for biomedical applications requiring high durability in physiological environments.

## Conclusions

This study successfully developed and characterized novel multiprincipal β-Ti alloys (Ti–33Nb–33Zr, Ti–25Nb–25Zr–25Ta, and Ti–40Nb–40Zr) for biomedical applications. All alloys exhibited a predominantly β-phase microstructure, with minor stress-induced α″ martensite and evidence of dynamic recrystallization. Although the ω phase was identified in the Ti–40Nb–40Zr alloy by TEM, its low volume fraction did not significantly affect the alloy’s low elastic modulus.

Among the investigated materials, Ti–40Nb–40Zr exhibited the lowest elastic modulus (42 GPa), approaching that of human bone, while Ti–25Nb–25Zr–25Ta presented the highest hardness and thermal stability. All alloys showed greater ductility than Ti–6Al-4 V, and β-transus temperatures below 500 °C, allowing efficient control of phase transformations through heat treatment.

Electrochemical analyses demonstrated excellent corrosion resistance in SBF for all alloys, with stable passive films and low corrosion current densities. The Ti–25Nb–25Zr–25Ta alloy exhibited the best electrochemical performance due to the formation of a Ta_2_O_5_-rich passive film, while the Zr-containing alloys also showed stable passivation through mixed TiO_2_–Nb_2_O_5_–ZrO_2_ oxides.

Overall, the developed multiprincipal β-Ti alloys combine low elastic modulus, high mechanical performance, and excellent corrosion resistance, making them promising candidates for next-generation load-bearing biomedical implants. These findings provide valuable guidelines for the design of advanced Ti-based biomaterials with improved mechanical compatibility and long-term stability under physiological conditions.
